# The Psychiatric Symptomology of Visual Snow Syndrome

**DOI:** 10.3389/fneur.2021.703006

**Published:** 2021-07-30

**Authors:** Emma J. Solly, Meaghan Clough, Paige Foletta, Owen B. White, Joanne Fielding

**Affiliations:** ^1^Department of Neuroscience, Central Clinical School, Monash University, Melbourne, VIC, Australia; ^2^Department of Neurology, Alfred Health, Melbourne, VIC, Australia

**Keywords:** visual snow syndrome, visual snow, depersonalisation, visual processing, sensory processing

## Abstract

**Objective:** To characterise the psychiatric symptoms of visual snow syndrome (VSS), and determine their relationship to quality of life and severity of visual symptoms.

**Methods:** One hundred twenty-five patients with VSS completed a battery of questionnaires assessing depression/anxiety, dissociative experiences (depersonalisation), sleep quality, fatigue, and quality of life, as well as a structured clinical interview about their visual and sensory symptoms.

**Results:** VSS patients showed high rates of anxiety and depression, depersonalisation, fatigue, and poor sleep, which significantly impacted quality of life. Further, psychiatric symptoms, particularly depersonalisation, were related to increased severity of visual symptoms. The severity/frequency of psychiatric symptoms did not differ significantly due to the presence of migraine, patient sex, or timing of VSS onset (lifelong vs. later onset).

**Conclusion:** Psychiatric symptoms are highly prevalent in patients with VSS and are associated with increased visual symptom severity and reduced quality of life. Importantly, patients with lifelong VSS reported lower levels of distress and milder self-ratings of visual symptoms compared to patients with a later onset, while being equally likely to experience psychiatric symptoms. This suggests that the psychiatric symptoms of VSS are not solely due to distress caused by visual symptoms. While no consistently effective treatments are available for the visual symptomology of VSS, psychiatric symptoms offer an avenue of treatment that is likely to significantly improve patient quality of life and ability to cope with visual symptoms.

## Introduction

Visual snow syndrome (VSS) is a neurological disorder characterised by a range of persistent visual disturbances. Its defining symptom is visual snow (VS), described as constant, flickering static across the entire visual field. Diagnosis of VSS requires the presence of VS for >3 months, alongside at least two of the following visual symptoms: palinopsia, enhanced entoptic phenomena, photophobia, and impaired night vision (nyctalopia) (1). For ~40% of patients, the visual disturbances associated with VSS have been present from their earliest memories; the remainder of patients experience a sudden or stepwise onset of symptoms, generally in the second or third decade of life. Symptom onset may be related to a migraine attack, but typically cannot be linked to a clear trigger (2, 3).

VSS often presents with a number of comorbidities, most commonly migraine and tinnitus (2, 4), but also other sensory symptoms, including paraesthesia and dizziness (5, 6). Notably, a range of psychiatric symptoms are consistently reported in VSS patients; these include depression and anxiety, (2, 3, 5), as well as fatigue, sleep disturbances (7, 8), and depersonalisation, a dissociative experience involving a sense of estrangement from the body (9). Irritability and difficulty concentrating are also frequent complaints (3). It is currently unclear how these psychiatric symptoms relate to each other or to the visual symptoms of the disorder, and ultimately how they impact quality of life. Since there is no cure or effective treatments available for the visual symptoms of VSS (10), management of psychiatric symptoms offers a viable means to alleviate the burden of VSS and improve patient quality of life.

Here, we comprehensively characterised the psychiatric symptomology of VSS in a large cohort of patients, using a combination of validated questionnaires and a structured interview. Specifically, we investigated the frequency of psychiatric symptoms and assessed their relationships to each other as well as to visual symptoms. In addition, we evaluated whether psychiatric symptom severity differed as a function of migraine status, onset of visual snow, and sex. Finally, we explored the impact of psychiatric symptoms on the frequency and severity of visual symptoms and their relative contribution to quality of life. The results of this research will provide important information for health professionals who encounter patients with VSS, and help identify treatment options allowing more effective management of the disorder.

## Methods

### Participant Recruitment

Participants with VSS were recruited primarily through online, radio, and television advertising, with a number of patients also referred by neurologists. Patients who had not been diagnosed with VSS by a neurologist were screened using an online questionnaire to confirm that they met the diagnostic criteria for VSS as specified by the International Classification of Headache Disorders (ICHD) (1). The questionnaire enquired about the onset and characteristics of the participant's VS, other visual symptoms the participant experienced, and whether they had ever undergone ophthalmological or imaging examinations regarding their VS.

Participants also were asked if they had been diagnosed with other ophthalmological, neurological, or psychiatric conditions, to exclude those with potentially confounding disorders. To exclude patients with Hallucinogen Persisting Perception Disorder (HPPD), which can present very similarly to VSS (11), participants were asked about illicit drug use. Those who reported illicit drug use in the 12 months preceding VSS symptom onset were considered possible HPPD patients and were not included in the study.

Forty-one healthy controls were recruited from the community through researcher social circles. Control data were used to determine normative ranges for the Short-Form 36 Health Survey global score and subscales, as clinical cut-off scores were not available. Exclusion criteria were a diagnosis of a confounding psychiatric, neurological, or ophthalmological condition. The mean age of controls was 27.2 (SD = 8.7): 15 (36.6%) male, and 26 (63.4%) female.

#### Standard Protocol Approvals, Registrations, and Patient Consents

Ethics approval was granted by the Monash University Human Research Ethics Committee. All participants provided written informed consent prior to participation in the study in accordance with the declaration of Helsinki.

### Measures

Participants completed a battery of online questionnaires relating to psychiatric symptomology and health-related quality of life. Standard methods for calculating scores and cut-offs for questionnaires were used unless otherwise indicated. In addition, VSS patients provided clinical information regarding age of VSS onset, VS symptom severity, presence of other symptoms (both visual and non-visual), and other relevant health information.

#### VSS Clinical Information

VS symptom severity was determined using a figure displaying varying intensities of “static.” Based on this figure, patients rated their VS intensity on a scale of 1–6, with 1 referring to the lowest intensity image and 6 to the highest intensity image. Patients also rated how disruptive they considered their VS to be on a scale from 1 (“Not at all disruptive”) to 7 (“Severely disruptive”), and how much they felt that VS has impacted their life on a scale from 1 (“No impact”) to 7 (“Severely reduced quality of life”). Further, they were given the option to list factors that improved or worsened their VS, and any life activities that were directly impacted by their visual symptoms.

A checklist of visual and non-visual symptoms commonly reported with VSS was provided to patients who were asked to nominate which symptoms, if any, they experienced. For symptoms patients may not be familiar with, such as depersonalisation and derealization, definitions were provided. Depersonalisation was defined as “feelings of being detached or disconnected from your body,” and derealization as “feelings that your surroundings or the people around you are not real.” Finally, patients reported whether they had ever been diagnosed with an anxiety or depressive disorder. Demographic and symptom information for VSS patients is presented in Table 1.

**Table 1:** Demographic information and symptom prevalence.

	**VSS patients (** ***n*** **=** **125)**	
	**Number**	**Percentage**
Age (mean, SD)	31.3, 10.4	–
Female (male)	63 (62)	50.4% (49.6%)
Lifelong VSS	45	36%
Later onset VSS:	80	64%
Age of onset (mean, SD)	22.4, 9.8	–
VSS duration (mean, SD)	9.7, 9.9	–
VSS intensity (mean, SD)	3.7, 1.3	–
VSS disruptiveness (mean, SD)	3.6, 1.6	–
VSS impact on quality of life (mean, SD)	3.7, 1.7	–
Migraine	61	48.8%
Family history of migraine	66	52.8%
Relative with VSS	5	4%
**Visual symptoms**		
Palinopsia: afterimages	108	86.4%
Palinopsia: trailing	66	52.8%
Nyctalopia	88	70.4%
Photophobia	58	46.4%
Floaters	113	90.4%
Blue field entoptic phenomena	91	72.8%
Halos	83	66.4%
Number of visual symptoms (mean, SD)	5.4, 1.8	–
**Non-visual sensory symptoms**		
Tinnitus	97	77.6%
Tremor	43	34.4%
Paraesthesia	59	47.2%
Dizziness	44	35.2%
**Other symptoms**		
Neck pain	63	50.4%
Irritability	70	56%
Concentration problems	99	79.2%
Depersonalisation	45	44.1%
Derealization	31	30.4%
Previous or current anxiety disorder	56	44.8%
Previous or current depressive disorder	48	38.4%

#### Depression Anxiety Stress Scale

The Depression Anxiety Stress Scale (DASS) is a commonly used measure of anxiety and depression (12) assessing self-reported negative emotions over the immediately preceding week. It consists of three scales: depression, anxiety, and stress, with higher scores reflecting higher levels of each symptom. Standard score ranges were used, as recommended by the DASS manual. Depression: Normal (0–9), Mild (10–13), Moderate (14–20), Severe (21–27), Extremely severe (28+). Anxiety: Normal (0–7), Mild (8–9), Moderate (10–14), Severe (15–19), Extremely severe (20+). Stress: Normal (0–14), Mild (15–18), Moderate (19–25), Severe (26–33), Extremely severe (34+).

#### Cambridge Depersonalisation Scale

The CDS measures self-reported experiences of depersonalisation over the previous 6 months (13). Higher scores indicate more frequent and severe depersonalisation, scores above 70 indicating clinical levels of depersonalisation (13).

#### The Pittsburgh Sleep Quality Index

The PSQI is a questionnaire assessing sleep quality over the past month (14). It consists of 7 subscales, each with a score range of 0–3: sleep quality, sleep latency, sleep duration, habitual sleep efficacy, sleep disturbances, use of sleeping medication, and daytime dysfunction. A global score ranging from 0 to 21 is also generated, with higher scores indicating poorer sleep. In the general population a global cut-off score of >5 is normally used, however in this study a more conservative cut-off score of >8 was chosen, as this has been suggested to be more appropriate in clinical populations (15).

#### The Fatigue Severity Scale

The FSS assesses the impact of fatigue on day to day functioning (16). It is a nine item self-report questionnaire requiring participants to respond on a scale of 1–7, with higher scores indicating higher levels of fatigue. Scores from each item were summed to form a total score ranging from 7 to 63. A cut-off score of >36 was used, equivalent to the cut-off score recommended by the original authors (16).

#### The Short-Form 36 Health Survey Version 2

The SF-36 is a commonly used measure of health-related quality of life comprising 8 subscales: physical functioning, role limitations due to physical problems, social functioning, bodily pain, mental health, role limitations due to emotional problems, vitality, and general health perceptions (17). Subscale scores are transformed to a scale from 0 to 100, with 0 representing the lowest possible score and 100 representing the maximum possible score. Higher scores indicate better health. A global score for the SF-36 was calculated to provide an overall indication of health-related quality of life, by summing the 8 raw subscale scores and similarly transforming to a 0–100 scale.

### Data Analysis

Data were analysed using SPSS statistics 27. Group means, standard deviations (SD), and the percentage of patients with scores falling above/below cut-offs were calculated for all questionnaires. Where published cut off scores were not available (SF-36 subscales and global score) control data were used by converting scores to z-scores based on the formula; (patient raw score – control population mean)/control population SD. For the SF-36, a z-score of −1.96 or below was considered indicative of significantly (*p* < 0.05) poor health relative to normative population scores. Z-scores were not calculated for the Pittsburgh Sleep Quality Index subscales due to the limited range of values for each subscale (0–3).

Where >25% of VSS patients scored outside defined normative cut-offs, analyses used independent samples *t*-test to determine whether psychiatric symptom severity differed due to the presence of migraine, patient sex, onset of symptoms (lifelong vs. later onset), or presence of depersonalisation. Chi square analyses were used to assess the likelihood of visual and non-visual symptoms occurring in each group. Relationships among questionnaire scores, number of visual symptoms, and VS self-ratings were examined using Pearson correlations, or Spearman correlations where appropriate. A backwards stepwise linear regression was run to assess the contributions of questionnaire scores to health-related quality of life (overall SF-36 score), and to VS intensity and the number of visual symptoms. An alpha-level of *p* < 0.05 was used to determine significance. Adjustments were not made for multiple comparisons.

## Results

Demographic information and prevalence of visual and non-visual symptoms in VSS patients are presented in Table 1.

**Figure 1:**
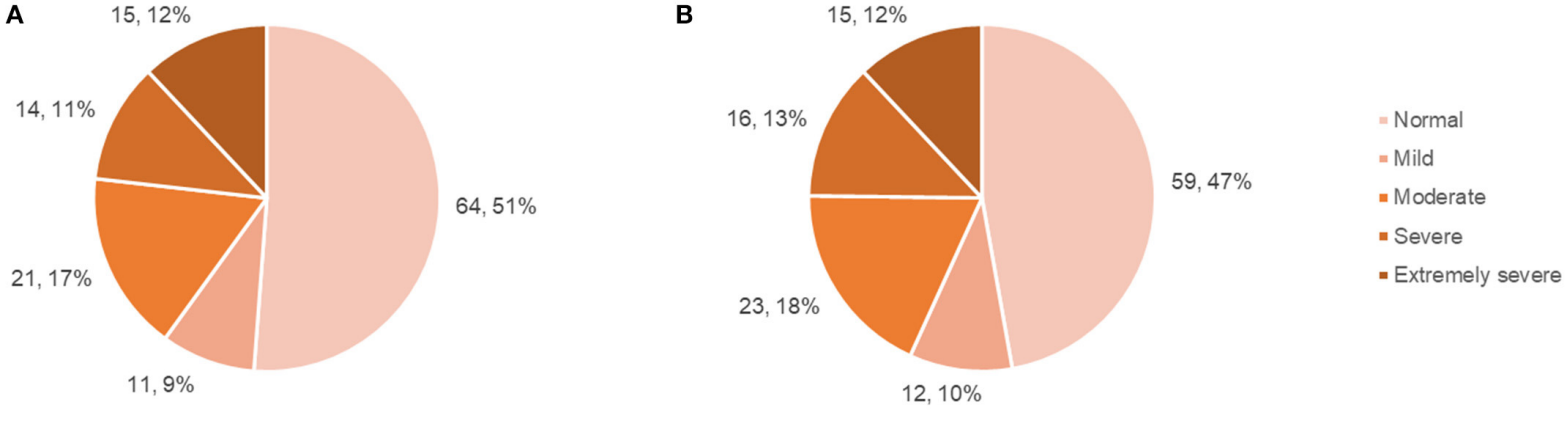
Patient DASS anxiety and depression subscale scores. **(A)** Number and percentage of patients falling within each DASS: anxiety score range, **(B)** Number and percentage of patients falling within each DASS: depression score range.

Questionnaire results (mean scores, SDs, number and percentage of patient scores falling above specified cut-offs) are presented in Table 2, with SF-36 results presented separately in Table 3. The number and percentage of patients in each DASS score range are displayed in Figure 1.

**Table 2:** Questionnaire results.

	**Mean (SD)**	**Scores above cut-off (%)**
**Depression Anxiety Stress scale**		
Depression	13.1 (10.9)	–
Anxiety	9.5 (8.4)	–
Stress	14 (9.6)	–
**Cambridge Depersonalisation Scale**	47.9 (41.1)	34 (27.2%)
**The Pittsburgh Sleep Quality Index**		
Global score	8.6 (3.7)	56 (44.8%)
Subjective sleep quality	1.3 (0.7)	–
Sleep latency	2 (1)	–
Sleep duration	0.8 (0.9)	–
Sleep efficiency	1.7 (1.4)	–
Sleep disturbances	1.4 (0.6)	–
Sleep medication	0.7 (1.1)	–
Daytime dysfunction	0.8 (0.9)	–
**Fatigue Severity Scale**	35.3 (15.1)	62 (49.6%)

**Table 3:** Short-Form 36 health survey results.

**Subscale**	**Mean (SD)**	**Significantly low scores (%)**
Global score	58.8 (16.1)	52 (41.6%)
Physical functioning	83 (21.9)	16 (12.8%)
Role: physical	67.9 (32.9)	47 (37.6%)
Role: emotional	48.4 (24.5)	41 (32.8%)
Bodily pain	48.2 (8.3)	3 (2.4%)
General health	52.9 (22)	31 (24.8%)
Vitality	36.2 (20.2)	31 (24.8%)
Social functioning	59.3 (29.2)	36 (28.8%)
Mental health	55.4 (20.2)	52 (41.6%)

### Sub-group Comparisons

#### Migraine

Psychiatric questionnaire scores did not differ between patients with and without migraine.

A higher number of visual symptoms was reported by patients with migraine (Mean = 5.9, SD = 1.7), relative to those without (Mean = 4.8, SD = 1.7), t_(123)_ = −3.6, *p* < 0.001, *d* = 0.1, 95% CI (−1.68, −0.49).

Patients with migraine were more likely to report photophobia [*X*${}_{\left(1,\text{N}=125\right)}^{2}$ = 7.63, *p* = 0.006], and palinopsia (visual trailing) [*X*${}_{\left(1,\text{N}=125\right)}^{2}$ = 5.93, *p* = 0.015]. The prevalence of other visual and non-visual symptoms did not differ.

#### Sex

Women exhibited higher levels of fatigue (FSS scores) than men (Mean = 39, SD = 14.2; Mean = 31.6, SD = 15.4), t_(123)_ = −2.8, *p* = 0.006, *d* = 0.5, 95% CI (−12.59, −2.12).

Women also rated the intensity of their VS as more severe (Mean = 3.94, SD = 1.13) than men (Mean = 3.42, SD = 1.35), t_(123)_ = −2.3, *p* = 0.022, *d* = 0.4, 95% CI (−0.96, 0.07). The prevalence of visual and non-visual symptoms did not differ.

#### VSS Onset

Psychiatric questionnaire scores did not differ between lifelong and later onset VSS patients.

Later onset VSS patients rated their VS as being more disruptive than lifelong VSS patients (Mean = 3.9, SD = 1.5; Mean = 3, SD = 1.5), t_(123)_ = 3.18, *p* = 0.002, *d* = 0.6, 95% CI (0.34, 1.44), with a greater impact on their quality of life (Mean = 4.13, SD = 1.69) than lifelong VSS patients (Mean = 2.82, SD =1.53), t_(123)_ = 4.27, *p* < 0.001, *d* = 0.8, 95% CI (0.69, 1.91).

Lifelong VSS patients were less likely to report palinopsia (visual trailing), *X*${}_{\left(1,\text{N}=125\right)}^{2}$ = 4.62, *p* = 0.032. The prevalence of other visual and non-visual symptoms did not differ.

#### Tinnitus

Psychiatric questionnaire scores did not differ between patients with and without migraine.

Patients with tinnitus reported a higher number of visual symptoms (Mean = 5.7, SD = 1.6), than those without tinnitus (Mean = 4.3, SD = 1.8), t_(123)_ = 0.33, *p* < 0.001, *d* = 0.8, 95% CI (−2.15, −0.75), and rated their VS as being more disruptive (Mean = 3.8, SD = 1.6), than those without (Mean = 2.9, SD = 1.3), t_(123)_ = −2.62, *p* ≤ 0.010, *d* = 0.6, 95% CI (−1.5, −0.21).

VSS patients with tinnitus were more likely to experience palinopsia (afterimages) [X${}_{\left(1,\text{N}=125\right)}^{2}$ = 10.56, *p* = 0.001], floaters [X${}_{\left(1,\text{N}=125\right)}^{2}$ =14.96, *p* < 0.001], and Blue Field Entoptic Phenomena (BFEP) [X${}_{\left(1,\text{N}=125\right)}^{2}$ =9.47, *p* = 0.002]. They also reported paraesthesia [X${}_{\left(1,\text{N}=125\right)}^{2}$ = 23.23, *p* = < 0.001], tremor [X${}_{\left(1,\text{N}=125\right)}^{2}$ = 6.47, *p* = 0.011], concentration problems [X${}_{\left(1,\text{N}=125\right)}^{2}$ = 4.87, *p* = 0.027], and neck pain [X${}_{\left(1,\text{N}=125\right)}^{2}$ = 6.89, *p* = 0.009], more often than patients without tinnitus.

Correlations between questionnaire scores, the number of visual symptoms, and self-ratings of VSS severity are presented in Table 4.

**Table 4:** Correlations between variables.

	**SF-36: Global**	**FSS**	**PSQI: Global**	**DASS: Depression**	**DASS: Anxiety**	**CDS**	**No. visual symptoms**	**VS Intensity**	**VS disruptiveness**	**VS impact on QoL**
SF-36: Global	–	−**0.484 (<0.001)**	**−0.373 (<0.001)**	**−0.619 (<0.001)**	**−0.653 (<0.001)**	**−0.559 (<0.001)**	−0.117 (0.194)	−0.151 (0.092)	**−0.204 (0.023)**	**−0.304 (<0.001)**
FSS	−0.**484 (<0.001)**	–	0.164 (0.067)	**0.276 (0.002)**	**0.299 (<0.001)**	**0.371 (<0.001)**	**0.270 (0.002)**	0.125 (0.165)	**0.186 (0.038)**	**0.217 (0.015)**
PSQI: Global	**−0.373 (<0.001)**	0.164 (0.067)	–	**0.265 (0.003)**	**0.446 (<0.001)**	**0.290 (<0.001)**	0.130 (0.148)	−0.014 (0.874)	0.008 (0.932)	0.017 (0.852)
DASS: depression	**−0.619 (<0.001)**	**0.276 (0.002)**	**0.265 (0.003)**	–	**0.596 (<0.001)**	**0.488 (<0.001)**	−0.021 (0.815)	0.005 (0.957)	0.068 (0.450)	**0.187 (0.036)**
DASS: Anxiety	**−0.653 (<0.001)**	**0.299 (<0.001)**	**0.446 (<0.001)**	**0.596 (<0.001)**	–	**0.473 (<0.001)**	**0.177 (0.048)**	0.069 (0.442)	**0.190 (0.034)**	**0.305 (<0.001)**
CDS	**−0.559 (<0.001)**	**0.371 (<0.001)**	0.**290 (<0.001)**	**0.488 (<0.001)**	**0.473 (<0.001)**	–	**0.256 (0.004)**	**0.288 (<0.001)**	**0.222 (0.013)**	**0.284 (0.001)**
No. visual symptoms	−0.117 (0.194)	**0.270 (0.002)**	0.130 (0.148)	−0.021 (0.815)	**0.177 (0.048)**	**0.256 (0.004)**	–	**0.209 (0.019)**	**0.455 (<0.001)**	**0.343 (<0.001)**
VS Intensity	−0.151 (0.092)	0.125 (0.165)	−0.014 (0.874)	0.005 (0.957)	0.069 (0.442)	**0.288 (<0.001)**	**0.209 (0.019)**	–	**0.496 (<0.001)**	**0.283 (0.001)**
VS disruptiveness	**−0.204 (0.023)**	**0.186 (0.038)**	0.008 (0.932)	0.068 (0.450)	**0.190 (0.034)**	**0.222 (0.013)**	**0.455 (<0.001)**	**0.496 (<0.001)**	–	**0.725 (0.001)**
VS impact on QoL	**−0.304 (<0.001)**	**0.217 (0.015)**	0.017 (0.852)	**0.187 (0.036)**	**0.305 (<0.001)**	**0.284 (0.001)**	**0.343 (<0.001)**	**0.283 (0.001)**	**0.725 (0.001)**	–

*Correlation coefficient (p-value). Bold, significant.*

*SF-36, Short Form 36 Health Survey; FSS, Fatigue Severity Scale; PSQI, Pittsburgh Sleep Quality Index; DASS, Depression Anxiety Stress Scale; CDS, Cambridge Depersonalisation Scale; VS, Visual snow; QoL, quality of life*.

### Relationship Between Psychiatric and Visual Symptoms

To estimate the proportion of variance in health-related quality of life (as estimated by the SF-36) that can be accounted for by fatigue (FSS), sleep (PSQI), depression (DASS: Depression), anxiety (DASS: Anxiety), and depersonalisation (CDS), we performed a multiple regression analysis using the stepwise backward elimination method. In combination, these variables accounted for a significant 60% of the variability in overall health-related quality of life (SF-36), *R*^2^ = 0.6, adjusted *R*^2^ = 0.59*, F*_(5, 120)_ = 45.49, *p* <0.001. Unstandardised (*B*) and standardised (β) regression coefficients for each predictor in the regression model are reported in Table 5.

**Table 5:** Quality of life regression analysis summary.

**Variable**	***B* (95% CI)**	**β**	***t***	***p***
FSS	−0.26 (−0.39, −0.13)	−0.24	−3.87	** <0.001**
PSQI: Global	–	–	–	–
DASS: Depression	−0.39 (−0.61, −0.17)	−0.27	−3.49	** <0.001**
DASS: Anxiety	−0.65 (−0.94, −0.37)	−0.34	−4.55	** <0.001**
CDS	0.07 (−0.13, 0.02)	−0.18	−2.57	**0.011**

*FSS, Fatigue severity scale; PSQI, Pittsburgh Sleep Quality Inventory; DASS, Depression Anxiety Stress Scale; CDS, Cambridge Depersonalisation Scale*.

Further multiple regression analyses using the stepwise backward elimination method were performed to determine the proportion of variance in the number of visual symptoms, and self-reported VS intensity, that can be accounted for by the same variables. In combination, these variables, excluding the PSQI which was again non-significant, accounted for 18% of the variability in the number of visual symptoms, *R*^2^ = 0.18, adjusted *R*^2^ = 1.5*, F*_(5, 119)_ = 6.42, *p* < 0.001. Unstandardised (*B*) and standardised (β) regression coefficients for each predictor in the regression model are reported in Table 6. Depression and depersonalisation accounted for 12% of the variability in VS intensity, *R*^2^ = 0.12, adjusted *R*^2^ = 0.12*, F*_(5, 119)_ = 8.58, *p* < 0.001, with the remaining variables non-significant. Unstandardised (*B*) and standardised (β) regression coefficients for each predictor in the regression model are reported in Table 6.

**Table 6:** Visual symptoms regression analysis summaries.

**Variable**	***B* (95% CI)**	**β**	***t***	***p***
**Number of visual symptoms**				
FSS	0.24 (0, 0.05)	0.21	2.35	**0.021**
PSQI: Global	–	–	–	–
DASS: Depression	−0.53 (−0.08, −0.02)	−0.33	−3.07	**0.003**
DASS: Anxiety	0.06 (0.13, 0.1)	0.27	2.54	**0.012**
CDS	0.01 (0, 0.02)	0.2	1.99	**0.049**
**VS intensity**				
FSS	–	–	–	**–**
PSQI: Global	–	–	–	–
DASS: Depression	−0.02 (−0.05, 0)	−0.21	−2.15	**0.034**
DASS: Anxiety	–	–	–	**–**
CDS	0.01 (0.01, 0.02)	0.4	4.14	** <0.001**

*FSS, Fatigue Severity Scale; PSQI, Pittsburgh Sleep Quality Inventory; DASS, Depression Anxiety Stress Scale; CDS, Cambridge Depersonalisation Scale*.

### Patient Experiences of Visual Snow

#### Factors That Worsened Visual Snow

Patients identified environmental and individual factors that worsened their VS. The most common environmental factors were dim or low-light conditions, harsh artificial light, bright sunlight, and darkness. The most common individual factors were tiredness/fatigue, stress/anxiety, alcohol consumption, inadequate sleep, exercise, caffeine, and screen use. Other factors mentioned less commonly included migraine, poor diet, dehydration, illness, menstruation, and illicit drug use (historically).

#### Factors That Improved Visual Snow

Most of the factors listed as improving VS were individual, with the most common, improving sleep, followed by “accepting” or learning to ignore symptoms (“looking through them rather than at them”), and improving diet. Other factors mentioned less commonly included altering ambient lighting, practising meditation or mindfulness, improving general mood, reducing stress/anxiety, and regular exercise. The patients who listed exercise as helpful clarified that exercise may intensify VS during physical activity, but led to improved perception of symptoms in the longer term. Patients frequently reported wearing sunglasses to reduce light sensitivity. Two patients reported wearing coloured lenses to reduce the perception of visual symptoms (5), and 2 reported that focusing on videos of static found online for 2–3 min decreased their VS significantly for ~30 s, although it did not provide long-lasting benefits.

#### Activities Impacted by Visual Snow

The most common activity impacted by VS was driving, with patients often specifying that driving at night/in the dark was difficult or impossible. Some patients elaborated on this, citing difficulty reading road signs, oncoming headlights being “too bright,” and afterimages of car lights and streetlights interfering with vision.

Patients also reported difficulty reading, with many adding that they avoided reading unless necessary. Issues using screens were also common, with one patient responding that he quit his career due to being unable to work on computers all day as required.

Other activities mentioned as impacted included social activities or sports which take place in bright daylight, going out at night, physical activity due to the temporary exacerbation of symptoms, and being able to enjoy natural scenery or stargazing. A number of patients simply replied broadly that their work, study, or social life were affected.

## Discussion

VSS remains a poorly understood disorder, with even less known about the frequently co-occurring psychiatric symptomology. Given the difficulty in treating the visual symptomology of VSS (10), understanding how psychiatric symptoms manifest and relate to the visual symptoms of the disorder offers avenues of treatment that may significantly improve quality of life for patients. Here we characterise the psychiatric symptoms most commonly reported by VSS patients, and their relationship with visual symptoms and quality of life (QoL). Our results show that patients with VSS more frequently exhibit clinically significant levels of depression, anxiety, depersonalisation, fatigue, and higher incidences of poor sleep, which significantly impact their QoL. Further, these psychiatric symptoms are related to more severe visual symptomology, with depersonalisation in particular consistently associated with more severe self-ratings of VS. Interestingly, the timing of VSS onset (lifelong vs. later onset), presence of migraine, and patient sex were not found to significantly impact the severity of psychiatric symptoms.

### Depression and Anxiety

Consistent with previous reports (3, 5, 8), a significant proportion of patients exhibited high levels of anxiety and depression, and poor overall mental health (SF-36: mental health subscale). DASS results indicated that 25% of patients exhibited either severe or extremely severe levels of depression, with a similar number exhibiting severe or extremely severe anxiety. Anxiety and depression scores were found to relate significantly to perceived VS severity. As with other neurological disorders, these symptoms might be assumed to reflect distress relating to the symptoms themselves, or may be linked to neurobiological changes underlying the specific disorder (18, 19). The impact of VSS visual symptomology on patient QoL can be profound, and is likely to contribute to higher levels of anxiety and depression. Key life activities including driving, reading, and screen use may be impacted, which frequently lead to difficulties with work and study; some patients stated that they changed employment or elected not to pursue further education as a result of their VSS. Social functioning also appears to be affected, with almost 30% of patients reporting significantly low scores on the SF-36 social functioning subscale. Social and recreational activities may be impacted by VSS in a number of ways: exercise, alcohol consumption, and bright or low-light conditions may temporarily exacerbate symptoms, resulting in avoidance of activities involving those factors. Some patients also reported that anxiety associated with visual symptoms affected their ability to leave their home.

In addition to the distressing nature and impact of their visual symptoms, there are many other factors influencing mental health. As VSS is still not widely recognised or understood, many patients struggle to find a diagnosis or explanation for their symptoms. Patients are often told that their symptoms are psychogenic, or presumed to be malingering (5, 20). Some patients involved in this study relayed having fears pre-diagnosis that they might have unidentified brain cancer, or that their visual symptoms would continue to progressively worsen until they became blind. Even following a diagnosis of VSS, most patients experience little if any relief from the few currently available treatments (10). These factors likely contribute to the prevalence of anxiety and depression in VSS patients.

However, our results indicate that anxiety and depression in VSS patients are not necessarily secondary to their disabling sensory symptoms, but may be, at least partially, inherent to the disorder. We anticipated that patients with lifelong VSS would report lower levels of anxiety and depression than patients with an onset later in life, given that for them, VS is “normal”; indeed, many lifelong patients report not realising their vision was abnormal until adulthood. In support of this, lifelong VSS patients rated their VS as being less disruptive and impacting their quality of life significantly less than patients with a later onset of symptoms. Yet despite being subjectively less concerned by their symptoms, lifelong VSS patients reported equal levels of depression and anxiety with later onset patients. The two groups also did not differ in sleep quality, level of fatigue, experiences of depersonalisation, or overall health-related quality of life. This suggests that the negative impact of VSS on mental health, sleep, and energy level is not solely attributable to distress caused by its symptoms.

### Sleep and Fatigue

Sleep difficulties have not been previously reported in VSS, however they were a frequent complaint among our cohort, and over 40% of patients exhibited sleep scale (PSQI) scores indicative of sleep dysfunction. Anecdotally, our VSS patients often reported difficulty sleeping due to the distracting and prominent nature of their visual symptoms in the dark, which are present with the eyes open or closed. We found that sleep difficulties were not limited to increased time to fall asleep (sleep latency), but also included high rates of sleep disturbances, poor sleep quality, and low sleep efficiency. As sleep reliably shows a bidirectional relationship with anxiety and depression (21), poor sleep may both contribute to and be influenced by poor mental health. Indeed, poor sleep was linked with higher rates of depression and anxiety. Given that many patients cited poor sleep as an aggravating factor for VS, addressing sleep difficulties in patients may lead to improvement in both mental health and the tolerability of visual symptoms.

Fatigue and lethargy have been previously reported in multiple VSS cohorts (3, 7). Interestingly, we did not find a significant relationship between sleep quality (PSQI) and level of fatigue (FSS), suggesting that the high levels of fatigue reported by VSS patients were not solely a consequence of poor sleep. Fatigue is highly prevalent in other neurological disorders, including migraine (22, 23), where it has been linked with more severe migraine symptomology and a greater level of impairment (22). Our results suggest a similar association in VSS, with increased levels of fatigue associated with a higher number of visual symptoms, and poorer self-ratings of VS severity. However, it remains unclear whether fatigue occurs as a consequence of coping with constant sensory dysfunction, whether patients with higher levels of fatigue are less able to ignore their visual symptoms and thus perceive them as worse, or whether fatigue is linked directly to the pathophysiology of VSS.

### Depersonalisation

Depersonalisation (CDS) scores showed the most consistent relationship with the number of visual symptoms and patient ratings of VS severity. CDS scores were also found to contribute significantly to VS intensity and the number of symptoms experienced. Depersonalisation refers to subjective feelings of detachment from the physical body and mental processes, and may involve a diminished sense of agency or feelings of being “robotic” (24). Temporary experiences of depersonalisation are common in the general population, but when they are recurrent and distressing a diagnosis of depersonalisation-derealization disorder may be considered (9). Nearly 45% of VSS patients in this study reported having experienced depersonalisation, and over 25% showed scores above the scale cut-off indicating a possible depersonalisation disorder. This suggests that depersonalisation is not only common in VSS, but is severe in a significant number of patients. Derealization, a dissociative experience characterised by a sense that the surrounding world is unreal or artificial (24), was also reported by ~30% patients. Derealization frequently co-occurs with depersonalisation; it is unclear whether they are truly distinct dissociative experiences, or whether derealization may represent a subtype of depersonalisation (25).

High rates of depersonalisation have been reported previously in patients with vestibular dysfunction (26, 27), retinal disease (28), and chronic dizziness (29). Here it has been theorised that feelings of depersonalisation may be evoked by a discrepancy between expected sensory input (provided by the frame of experience) and actual (aberrant) sensory experience (28); or, alternatively, by a discrepancy between signals from different sensory systems due to one or more being disrupted (30). A coherent perception of the body and surrounding space requires the seamless integration of inputs from different sensory modalities; conceivably, disturbance of this integration may lead to a disrupted awareness of the self (30). As depersonalisation and derealization are not well-known as symptoms, or as a disorder, patients may lack the language to describe their experiences, or may not realise what they are experiencing is a defined psychological symptom. Health professionals may therefore need to describe these experiences to a patient to ascertain whether they have experienced them.

### Pathophysiology of VSS

Although it is unclear whether poorer mental health and difficulties with sleep and fatigue are primary VSS symptoms, or secondary to sensory dysfunction, a shared underlying cause is plausible. The pathophysiology of VSS remains unknown, but is theorised to involve a central disturbance in the processing of sensory information (3, 5, 20). In our previous studies investigating ocular motor performance in VSS patients (6), we identified attentional changes hypothesised to be consistent with disrupted thalamocortical (TC) communication, potentially a thalamocortical dysrhythmia (3, 5). With the exception of the olfactory system, all sensory input passes through the thalamus, which regulates the incoming flow of sensory information to the cortex (31, 32). The thalamus and cortex are highly interconnected through recurring feedback loops. TC circuits are characterised by state-dependent resonant oscillatory activity, which modulates thalamic and cortical functioning (33). The oscillatory activity of TC networks is crucial not only for the processing and integration of sensory information, but also for attention, cognition, and arousal level (34, 35). As such, alteration in the oscillatory activity of TC networks may conceivably underlie the range of sensory and non-sensory symptoms reported by VSS patients, including disrupted sleep and fatigue. Evidence also exists that dissociative experiences may involve abnormal TC network activity (36).

Thalamocortical dysrhythmia (TCD) is a form of aberrant TC oscillatory activity proposed to underly a number of neurological and psychiatric disorders including migraine, tinnitus, and depression (37–40). In TC networks, different behavioural states are characterised by specific forms of oscillatory activity (34). TCD involves a pathological increase in low-frequency theta waves during states of wakefulness, coupled with surrounding high-frequency gamma waves (37, 40). These oscillatory changes result in disruption to normal state-dependent communication between the thalamus and cortex, which may manifest as a diverse range of affective, cognitive, and sensory symptoms, depending on which TC networks and associated cortical areas are implicated (40, 41). TCD occurs due to increased inhibitory or decreased excitatory input at the thalamic level, which may be triggered by bottom-up or top-down processes (37). It has been theorised that TCD in VSS may be secondary to cortical excitability (5).

Potential limitations of this study include recruitment bias, and a lack of objective measures of VSS severity. Patients who are more impacted by their symptoms may be more motivated to seek out and engage in research, biassing studies toward reporting more severe cases. Self-ratings of VS are also unlikely to be purely objective, reflecting distress relating to symptoms as well as symptom severity. Correlations between self-ratings of VS and questionnaire scores should therefore be interpreted with the awareness that they may reflect both the impact of VS on quality of life, and the influence of psychiatric symptomology on the perception of symptoms. Indeed, many patients reported that changes in psychiatric symptomology (e.g., in anxiety level or sleep quality) worsened or improved their perception of visual symptoms.

### Treatments

Treating the psychiatric symptomology associated with VSS is likely to significantly improve patient QoL, with or without accompanying improvement in visual symptoms. Sleep dysfunction is highly treatable through a number of approaches including lifestyle changes, behavioural therapies, and pharmacological agents (42). Addressing sleep dysfunction may also lead to improvements in depression and anxiety due to the bidirectional relationship between sleep and mental health (21). Identifying and treating patients at risk of severe mental health problems, and possibly suicidality, is also highly important given the rates of severe depression and anxiety associated with VSS. As with sleep dysfunction, a number of treatment avenues are available for anxiety and depression, both pharmacological and psychological (43, 44). While little research has been conducted into treatments for depersonalisation, there is some evidence that lamotrigine, currently the medication considered most efficacious in VSS (10), may be helpful in treating this symptom (45). Psychological therapies commonly used to treat depression and anxiety, such as cognitive behavioural therapy (CBT) may also help patients cope with their visual symptoms.

No study to date has investigated psychological approaches to treating VSS; however, a wealth of research exists on CBT based treatments for tinnitus. Tinnitus is reported by the majority of VSS patients, and has been theorised to represent an auditory analogue of VS; i.e., the constant perception of low level “noise” in each sensory system (3). Given the similarities between tinnitus and VS, and overlap of patients, treatments shown to be effective for tinnitus may also be helpful in VSS. CBT encompasses a wide variety of cognitive and behavioural therapeutic techniques, but simplistically, typically aims to identify and modify negatively biassed or irrational reactions to events and experiences, such as the perception of tinnitus (or VS) (46). CBT has been shown to significantly improve depression, anxiety, insomnia, and overall health-related QoL in tinnitus patients (47). Forms of CBT developed to treat tinnitus may be efficacious if adapted for use in VSS.

## Conclusion

Our results show that VSS significantly impacts a patient's QoL, affecting various aspects of physical and mental health. Anxiety and depression, depersonalisation, disrupted sleep, fatigue, and impaired social functioning were highly prevalent among patients, with poorer scores on these variables typically relating to worse self-ratings of visual symptoms. Although the equivalent QoL scores of lifelong and later onset patients indicate that mental health, fatigue, and sleep difficulties may be partially inherent to the disorder, they are doubtless exacerbated by the emotional impact of sensory symptoms. Patients reported that poor sleep, tiredness, and anxiety worsened their VS, whereas improving sleep and reducing stress and anxiety were said to improve perception of symptoms. Given the sparsity of effective treatments available for VSS, treating associated mental and physical health symptoms may in some cases be the only and most effective method of assisting patients. Managing the mental health and sleep of patients is likely to improve their overall quality of life and lead to improvement in the perception of visual symptoms.

## Data Availability Statement

The raw data supporting the conclusions of this article will be made available by the authors, without undue reservation.

## Ethics Statement

The studies involving human participants were reviewed and approved by Monash University Human Research Ethics Committee. Written informed consent to participate in this study was provided by the participants.

## Author Contributions

JF, MC, OW, ES, and PF contributed to the conception and design of the study. Acquisition of data was handled by ES and PF. ES wrote the first draft of the manuscript. JF, MC, and OW supervised the project and were involved in critical revision of the manuscript. All authors contributed to the article and approved the submitted version.

## Conflict of Interest

OW receives discretionary research funding from Merck, and is an Associate Editor for Frontiers in Neuro-opthalmology. JF receives funding for other research from Biogen and Genzyme and VSI. The remaining authors declare that the research was conducted in the absence of any commercial or financial relationships that could be construed as a potential conflict of interest.

## Publisher's Note

All claims expressed in this article are solely those of the authors and do not necessarily represent those of their affiliated organizations, or those of the publisher, the editors and the reviewers. Any product that may be evaluated in this article, or claim that may be made by its manufacturer, is not guaranteed or endorsed by the publisher.
